# Genome-Wide Transcriptome and Binding Sites Analyses Identify Early FOX Expressions for Enhancing Cardiomyogenesis Efficiency of hESC Cultures

**DOI:** 10.1038/srep31068

**Published:** 2016-08-09

**Authors:** Hock Chuan Yeo, Sherwin Ting, Romulo Martin Brena, Geoffrey Koh, Allen Chen, Siew Qi Toh, Yu Ming Lim, Steve Kah Weng Oh, Dong-Yup Lee

**Affiliations:** 1 Bioprocessing Technology Institute, Agency for Science, Technology and Research (A*STAR), 20 Biopolis Way, #06-01, Singapore 138668, Singapore; 2 Department of Chemical and Biomolecular Engineering, National University of Singapore, 4 Engineering Drive 4, Singapore 117585, Singapore; 3 USC Epigenome Center, University of Southern California, Los Angeles, CA 90033, USA; 4 NUS Synthetic Biology for Clinical and Technological Innovation (SynCTI), Life Sciences Institute, National University of Singapore, 28 Medical Drive, Singapore 117456, Singapore

## Abstract

The differentiation efficiency of human embryonic stem cells (hESCs) into heart muscle cells (cardiomyocytes) is highly sensitive to culture conditions. To elucidate the regulatory mechanisms involved, we investigated hESCs grown on three distinct culture platforms: feeder-free Matrigel, mouse embryonic fibroblast feeders, and Matrigel replated on feeders. At the outset, we profiled and quantified their differentiation efficiency, transcriptome, transcription factor binding sites and DNA-methylation. Subsequent genome-wide analyses allowed us to reconstruct the relevant interactome, thereby forming the regulatory basis for implicating the contrasting differentiation efficiency of the culture conditions. We hypothesized that the parental expressions of FOXC1, FOXD1 and FOXQ1 transcription factors (TFs) are correlative with eventual cardiomyogenic outcome. Through WNT induction of the FOX TFs, we observed the co-activation of WNT3 and EOMES which are potent inducers of mesoderm differentiation. The result strengthened our hypothesis on the regulatory role of the FOX TFs in enhancing mesoderm differentiation capacity of hESCs. Importantly, the final proportions of cells expressing cardiac markers were directly correlated to the strength of FOX inductions within 72 hours after initiation of differentiation across different cell lines and protocols. Thus, we affirmed the relationship between early FOX TF expressions and cardiomyogenesis efficiency.

Cardiomyocytes that are differentiated in a *de novo* manner from induced pluripotent stem cells have tremendous applications in the repair of damaged heart muscle^1^,^2^, understanding of disease progression^3^, drug efficacy screening^4^ and cardiac toxicity tests^5^. The efficacy of the process (cardiomyogenesis) depends on applied protocol^6^, cell line propensity^7^,^8^, and its epigenetic memory^9^,^10^ which becomes heterogeneous population-wise with increasing passage numbers^11^. In view of these challenges, much effort has been put into understanding and resolving them. For instance, researchers have characterized cell lines according to their inclination to convert into the mesoderm lineage from which cardiac cells can be derived. They generated reference ‘omics’ maps based on standard profiles in order to extrapolate the differentiation potential of other cell lines^12^. To further reduce the need for expensive and time-consuming experiments, a predictive epigenetic biomarker was also employed to identify cells with reduced differentiation capacity^13^. In addition, to directly evaluate and control cardiomyogenesis progression, accurate and reliable molecular assays were also developed. For instance, *NCAM1*+/*EpCAM*- expression profile was used to mark epithelial-to-mesenchymal transition^14^ during initiation of differentiation while subsequent generation of mesodermal cells was confirmed by *T* and *MIXL1* expressions^15^,^16^. There were also genes defining cardiac-mesoderm specification (*MESP1*^17^ or *KDR*+/*PDGFRA*- expression profile^18^) and the final cardiomyocytes (*GATA4, TBX5, NKX2-5* and *MEF2C*^6^). After differentiation, ‘omics’-based algorithms are now available for predicting the fidelity of resulting cells^19^. Other developments were related to cell cultures and differentiation protocols. For instance, next-generation Matrigel-based platforms have been introduced to avoid cross-species contamination which has been an issue with hESCs grown on mouse embryonic fibroblast (MEFs) feeders. Correspondingly, molecularly-defined protocols, e.g., CHIR99021/IWR1, suited for Matrigel cultures have been advanced^20^. They often require timely activation of WNT and TGF-beta signaling pathways, followed by in-activation in order to induce cardiac specification^21^,^22^. However, with the rapid development in cell lines, cultures and differentiation protocols, there is a lack of understanding on their interplay which renders it difficult to consistently achieve high cardiomyogenesis efficiency.

In this study, we investigated such interactions by comparing hESCs grown in three different MEF- and Matrigel-based cultures (Fig. 1). Specifically, in addition to mouse feeders and Matrigel, we also designed an intermediate condition with cells grown on Matrigel then replated on MEFs. Initially, we quantified their contrasting cardiomyogenesis efficiency based on a SB203580^23^ differentiation protocol that has worked well for MEF-based cultures but not Matrigel. We then profiled and analysed their regulation differences in the context of the transcriptome, DNA-methylome and transcription factor (TF) binding-sites. By integrating genome-wide ‘omics’ and knowledge-based analyses, we elucidated critical changes in signaling pathways and identified downstream regulators that are quantitative indicators of cardiomyogenesis potential during early differentiation. We verified the utility of such markers in the differentiation of two cell lines under Matrigel condition using a different protocol. Finally, we spelt out the fundamental factor underlying cell lines, culture conditions and protocols which determine cardiomyogenic outcome.

## Results

### Phenotypic, genetic and epigenetic profiling of distinct hESC cultures

We analyzed the effect of various culture conditions on the cardiomyogenesis efficiency of HES-3 cells. Cells were grown on (1) Matrigel-coated surfaces (MGEL-cells), (2) MEF feeders (MEF-cells) and (3) Matrigel replated onto feeders (RPL-cells) (Methods). Upon treatment with a protocol^24^ based on SB203580^23^ small molecule, the proportion of beating cell aggregates was evaluated as a proxy measure of differentiation. Expectedly, the final populations originating from MEF-cells had 86% beating aggregates while RPL-cells and MGEL-cells led to 54% and 0%, respectively (Fig. 2a). Apparently, SB203580 molecule effectively induced differentiation of MEF-cells by overcoming a cardiac differentiation barrier^6^. However, the relatively low and even zero differentiation of respective RPL-cells and MGEL-cells implicated the presence of unidentified molecular bottleneck(s) leading to their poor outcomes (Fig. 2b). In order to confirm molecularly that the beating aggregates were indeed cardiomyocytes, they were cryo-sectioned and affirmatively stained with antibodies against a cardiac marker troponin-T (Fig. 2c, red stain).

To decipher the transcriptional basis for the hypothesized bottleneck, we profiled cellular gene expressions using microarray. We reasoned that expression changes in RPL-cells compared to MEF-cells should account for the decrease in cardiomyogenesis efficiency from 86 to 54% (Fig. 2a) while a further decrease from 54% to 0% efficiency lies in the comparison between RPL-cells and MGEL-cells; a complementary understanding was also derived from comparing MEF-cells (86%) to MGEL-cells (0%). After comparative analyses^25^ (Fig. 2d), we pooled together differentially-expressed genes (DEGs) and clustered them to uncover gene dynamics modulating cardiomyogenesis (Fig. 2e). We looked for and identified a class of genes consistently changing in its expression (MGEL baseline) from MEF-cells to RPL-cells to MGEL-cells, following the trend in cardiomyogenesis efficiency (left panel, Fig. 2e). We speculated that such gene regulation may provide the basis for explaining the disparate differentiation potential among the cell cultures. Since gene expressions can be affected by epigenetic changes during passaging and re-plating^11^, and may have crucial effects on cardiomyocyte development^26^, we also profiled and analysed the DNA-methylation accordingly. Further analyses showed that the expression profiles of specific genes following the trend in cardiomyogenesis (left panel, Fig. 2e) can be explained by similar DNA-methylation dynamics (Supplementary information).

### Functional analysis uncovered increased mesodermal expressions in MEF-cells

We screened the biological functions of genes with consistently increasing or decreasing expressions in the direction: MGEL-cells → RPL-cells → MEF-cells (left panel, Fig. 2e), which could explain similar cardiomyogenesis propensity ranking. Importantly, a quarter (43/182) of these genes were involved in developmental processes, e.g., those of the mesoderm (P-value = 1.3E-04, enrichment fold change [FC] = 3.05) and ectoderm, suggesting a shift in differentiation capacities merely due to culture conditions (Fig. 3a). However, we were quick to discern that cardiac, cardiac-muscle differentiation and development genes were not likewise enriched (P-values > 0.05). Thus, by understanding cardiomyogenesis as consecutive mesoderm and cardiac differentiations (Fig. 2b), we can infer that the cardiomyogenesis capacity of MEF-cells may be promoted by its highest mesodermal expressions among the cell cultures rather than cardiac-related genes. Based on paired T-tests on three mesodermal gene-sets (Methods), we affirmed mesodermal up-regulation in MEF-cells, compared to either RPL-cells or MGEL-cells that have lower cardiomyogenesis efficiencies. The associated p-values were significant, being less than 5% for all comparisons (Fig. 3b). Dovetailing with our hypothesis that mesodermal genes were determinants, again there was no up-regulation for cardiac gene-sets (p-values > 0.05), in addition to three other findings that precluded cardiac programs (Supplementary Information). All these results motivated us to find more evidence of mesodermal DEGs enhancing cardiomyogenesis.

Among implicated mesodermal candidates (Table 1), almost 2/3 (11/18) were already reported to be associated with cardiomyogenesis or cardiovascular development. For instance, the mesoderm morphogen WNT3A is known to induce cardiac differentiation of ES cells^27^; NODAL-dependent signaling promotes cardiomyogenesis over neural differentiation of ES cells^28^. In searching for underlying transcriptional regulators, we collectively identified 6 FOX TFs that were present in an enriched manner, and hence co-regulated with the gene class putatively (P-value = 1.21E-5, enrichment FC = 20.54) (Fig. 3a). Of these TFs, only FOXC1, −D1 and −Q1 significantly regulated the gene class, with their targets having enrichment p-values ranging between and 0.002 and 0.033, covering 59% (107/182) of all genes. It should be highlighted that although FOXC1 is known to be involved in cardiovascular development^29^, myogenesis^30^, and mesoderm development^31^, a total of three TFs, i.e. FOXC1, −D1 and −Q1, were newly hypothesized regulators of the cardiomyogenic propensity of hESCs. The finding was partially supported by the widespread roles of the TF family in mesoderm development^31^. On the other hand, functional analyses of the other two regulation classes (right panel, Fig. 2e) primarily revealed (i) defence and immune responses to re-plating and (ii) regulation of cell death and proliferation that were not directly related to cardiomyogenesis tendency (Supplementary Data).

### Mesodermal interactome as basis for contrasting differentiation capacity of hESC cultures

To further shed light on core factors promoting differentiation, we leveraged on interaction data by building a relational model based on expert knowledge and verified mechanisms of cardiomyogenesis, binding site data of FOX TFs and other human-specific molecular interactions (Methods). Consistently, we identified a DEGs-enriched hub in which the trio TFs have binding sites on themselves and each other coupled with auto- and mutually correlated expressions, which are also in line with differentiation outcome (Fig. 4). Potentially pointing to their related functional roles, FOXC1 and -D1 have similar DNA specificities, and thus possibly regulate genes with redundancy^32^. If true, such a mechanism could have either fine-tuned or ensured stability of co-target expressions including themselves. Among the FOX’s correlated targets, WNT3 and EOMES, like FOXC1, are potent enhancers of cardiomyogenesis^27^,^33^ by generating differentiation cascades involving BMP4, T-bra^6^, NKX2-5 and MESP1^33^. The latter’s activation downstream of FOXC1 was also suggested from the observation that its expression was lost in the presomitic mesoderm without FOXC1^34^. Upstream of these TFs, our interactome model revealed the FOX TFs receiving inputs from WNT^30^, NODAL, FGF and Hedgehog pathways, thus indicating how feeder-induced signaling in MEF-cells may increase mesodermal expressions and hence promote cardiomyogenesis. Similar signaling mechanisms for FOX regulation were described in other contexts^31^.

We interrogated signaling mechanisms for regulating FOX TFs in hESCs. WNT, TGF-beta, Hedgehog and MAPK pathways were assessed to be genetically ‘rewired’ with their gene enrichment −log_10_ P-values ranging between 2.8–10.5 at 15.5–55.9 times over-representation for DEGs (inner panel, Fig. 5). Thus, we surmised that the HES-3 cultures had different signaling sensitivity. WNT was the most significantly enriched in DEGs, and positive signaling effectors in the pathway either had decreased negative regulation (FZD3, −4, −7, −8, FRAT2, AXIN2, SMAD4 and EP300) or increased positive regulation (WNT3) in MEF-cells (outer panel, Fig. 5), thus consistently promoted signaling. To uncover their regulation, specific components promoting cardiomyogenesis (WNT3^27^, ID3^35^ and ZIC2^36^) were further determined as putative targets of the FOX TFs (Table 2). As a rough gauge to quantify mouse feeder-induced signaling, we evaluated the relative capacity of WNT and 4 other cascades in MEF- over RPL-cells (Table 3). After multiplying the log_2_FC regulation of each signaling molecule with its effect on the downstream substrate (positive effector: 1; negative effector: −1), a resulting series of large positive numbers for consecutive cascade molecules would suggest stronger and more consistent regulation, and improved cardiomyogenesis outcome. In this manner, cascades starting with WNT3, BMP, NODAL, FGF2 and ZIC2 activators and ending in mesoderm induction or FOX’s activation were determined to have consistently high positive numbers for MEF-cells over RPL-cells. Of the 5 cascades, WNT3 had the most contributing molecular components (6 in Table 3), and its augmented signaling potential in MEF-cells (mean log_2_FC = 1.03, Table 3) suggested it as a plausible avenue for FOX activation.

### WNT-induced FOX expressions tied to cardiomyogenesis efficiency of hESCs platforms

**Figure 1:**
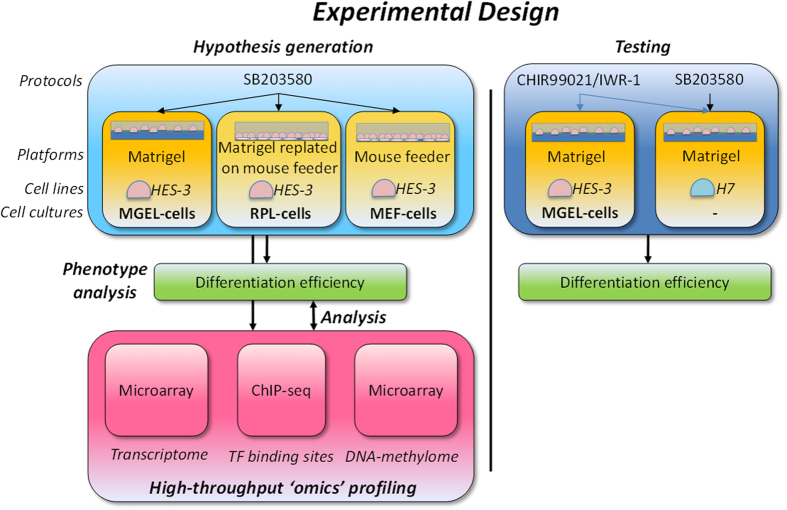
Experimental design involving different cell lines, culture platforms and differentiation protocols. Regulatory factors determining cardiomyogenesis efficiency are identified by profiling differentiation efficiency, transcriptome, TF binding sites and DNA-methylation, and their genome-wide analyses. The hypothesis generated under designed conditions is then tested in a different set of conditions.

**Figure 2:**
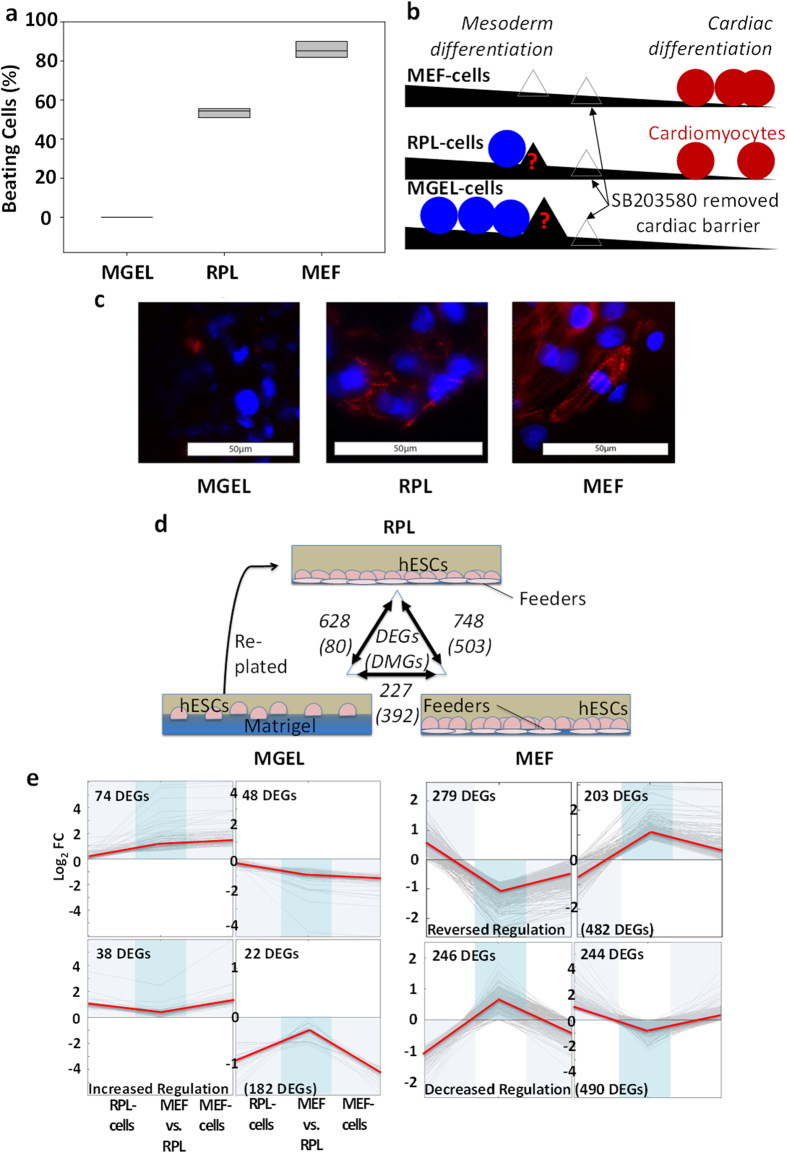
Phenotypic, genetic and epigenetic profiling of hESC cultures. (**a**) Percentage of beating aggregates, 12 days after applying SB203580 protocol. Biological triplicates were used. (**b**) Cardiomyogenesis model in MEF-cells versus MGEL-cells. Arrowed empty triangles: Hypothesis of cardiac differentiation ‘road block’ being removed by SB203580 protocol during cardiomyogenesis of HES-3 cultures; filled triangles: additional unidentified bottleneck(s) in RPL-cells and MGEL-cells but removed in MEF environment. (**c**) Embryoid bodies from the three hESC cultures were stained with troponin-T antibody (red) 12 day after application of the same protocol. The nuclei were stained with DAPI (blue). Scale bars of 50 microns were marked in the images. (**d**) Number of differentially-expressed genes (DEGs) and differentially-methylated genes (DMGs) between HES-3 cultures. (**e**) Gene expression dynamics after K-means clustering using dot-product metric. Log2 fold change level of RPL-cells and MEF-cells was in comparison to MGEL-cell baseline.

**Figure 3:**
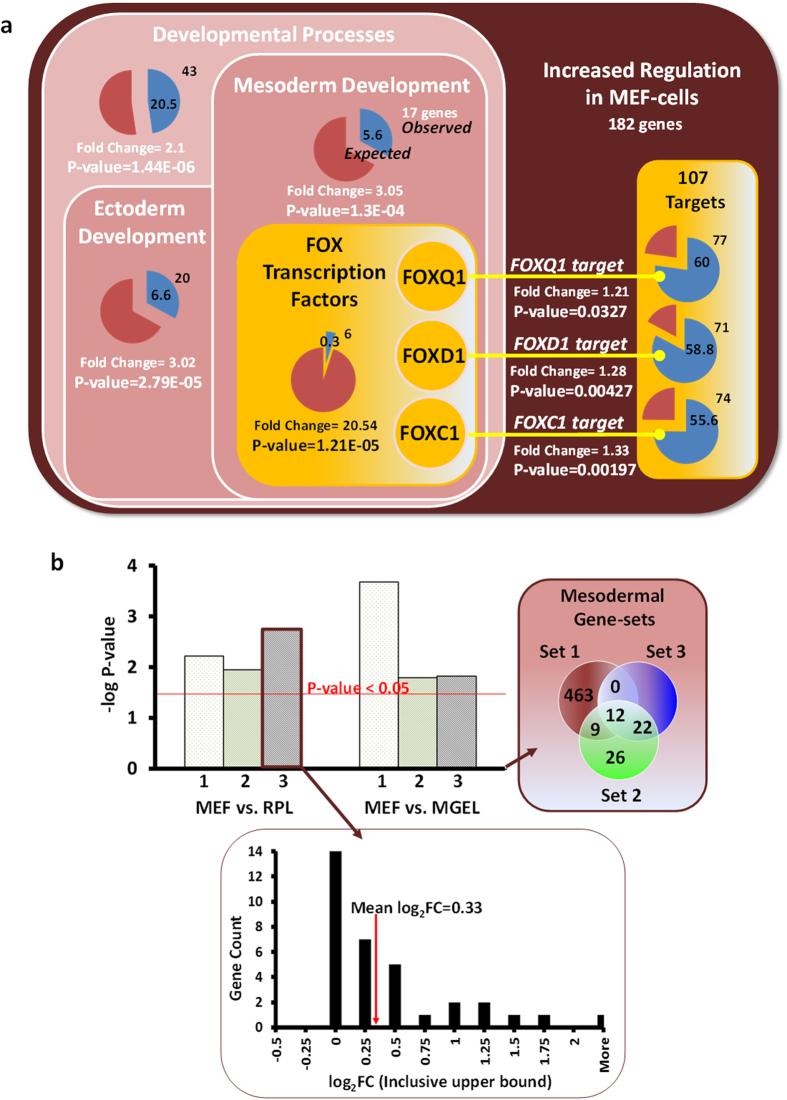
Functional and transcription factor binding site analyses of genes with increased regulation in MEF-cells compared to RPL-cells. (**a**) Significant enrichment of developmental genes, FOX TFs and their transcriptional targets. (Refer to method for definition of ‘increased regulation’.) (**b**) −log P-values of paired t-tests comparing MEF-cells with RPL-cells, and MEF-cells with MGEL-cells based on three definitions of mesodermal gene-sets (Methods). Bottom panel example: log_2_ fold change (FC) histogram of mesodermal gene-set 3 comparing MEF-cells to RPL-cells.

To demonstrate FOX inductions by WNT activation, we measured their expressions in MGEL-cells after treatment with a WNT-inducing CHIR99021/IWR-1 protocol^37^. The TFs and their supposed targets, EOMES and WNT3, were indeed activated in the first 72 hours (left panel, Fig. 6a). Note that such activations were much less with SB203580^23^,^38^ treatment whereby no beating aggregates were observed (Fig. 2a). Consistently, fluorescence-activated cell sorting (FACs) analysis on day 12 of differentiation upon CHIR99021/IWR-1 treatment, gave rise to a higher 93.4% of cells testing positive for cTnT expression, compared to just 5.1% after SB203580 treatment (Top panel, Fig. 6b). Similar proportions tested positive for the expression of another cardiac gene, MLC-2A (93.0% for CHIR99021/IWR-1 and 10.8% for SB203580), affirming the higher cardiomyogenesis efficiency of CHIR99021/IWR-1-treated, FOX-activated cells. We repeated CHIR99021/IWR-1 and SB203580 treatments on a different H7 cell line (Fig. 1), resulting in similar observations with regard to their relative FOX, EOMES and WNT3 activations (right panel, Fig. 6a) and cell proportions tested positive for both cardiac markers (bottom panel, Fig. 6b).

Between HES-3 and H7 cells, the timings, spans and maximum intensities of FOX induction were expectedly dissimilar due to embryonic differences in their genetic and signaling pathways. However, maximum induction is indicative of cardiomyogenesis efficiency; to demonstrate the highly linear relationship between FOX induction in the first 72 hours and final differentiated-cell proportion (day 12), we reported their Pearson correlation coefficients ranging between +0.85 and +0.98 (Table 4). Among them, FOXQ1 had the largest correlation with a one-tailed P-value of 0.009, in spite of the small sample size involved (n = 4). Within its linear range, a 39.4X induction was sufficiently associated with 100% of cells expressing cTnT cardiac marker (Fig. 6c). To appreciate the importance of maximum induction, we noted it as a population-average that was informative of cell number crossing decision threshold (to become beating aggregates in our context) in a bistable system. The latter is commonly found in biological networks with positive feedback loops. Even if the decision threshold is attained temporarily in a sub-population, cellular memory of the event (hysteresis)^38^ commits affected cells to their destined outcomes in different biological processes^39^,^40^,^41^,^42^,^43^ including differentiation. In this sense, we understood why hESCs may only respond to maximum FOX TFs’ fold-changes for cardiomyogenesis induction (Fig. 6c) but not necessarily their precise timing and span. While our data was consistent with the idea of positive feedbacks among the trio driving differentiation (Fig. 6d), we hastened to emphasize the current lack of evidence for such a mechanism.

We further clarified the relationship between parental FOX expressions and cardiomyogenesis efficiency (Fig. 6e). The TFs were up-regulated in both MEF-cells and RPL-cells compared to MGEL baseline using qRT-PCR analysis; MEF-cells’ expression levels were also more than two folds the level in RPL-cells, which was consistent with their relative cardiomyogenesis efficiency (blue region in Fig. 6e). Their implicated target, WNT3, had a similar expression profile while other FOX TFs were not associated with cardiomyogenesis efficiency (outside blue region in Fig. 6e). In a nutshell, we made evident that FOX levels were significant indicators of cardiomyogenesis outcome in different cell lines, culture platforms and differentiation protocols.

## Discussion

**Figure 4:**
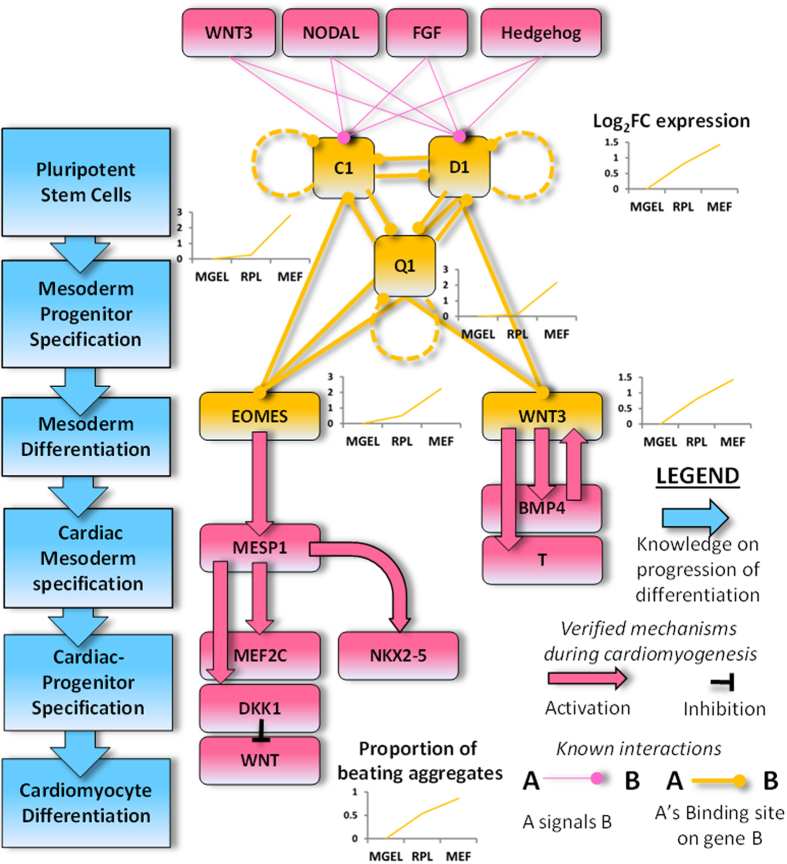
Summary of the relationships among FOX TFs and related factors mediating cardiomyogenesis. The gene expression profiles of FOX TFs, EOMES and WNT3 in hESCs under three cell culture conditions were correlated with final proportion of beating aggregates.

**Figure 5:**
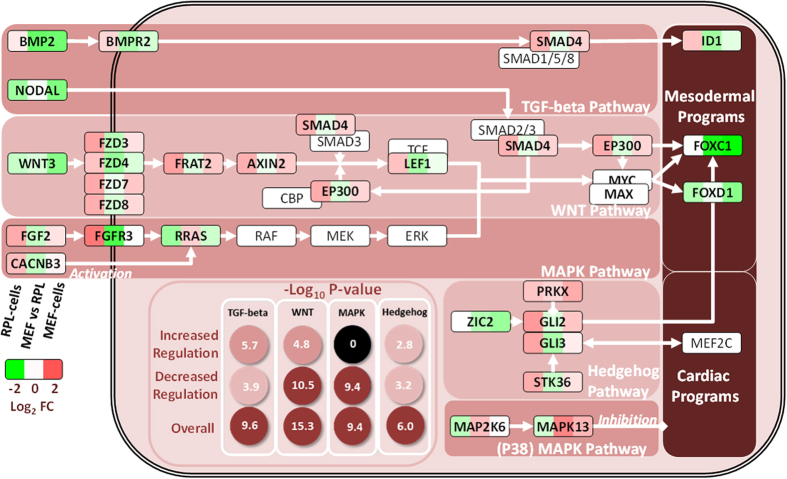
Signaling pathways implicated in cardiomyogenesis. Inner panel: Gene enrichment P-values according to gene dynamics. (Refer to method for definition of ‘increased regulation’ and ‘decreased regulation’.) Outer panel: Signaling cascades identified from reconstructed interactome that potentially enhanced cardiomyogenesis in MEF-cells compared to RPL-cells.

**Figure 6:**
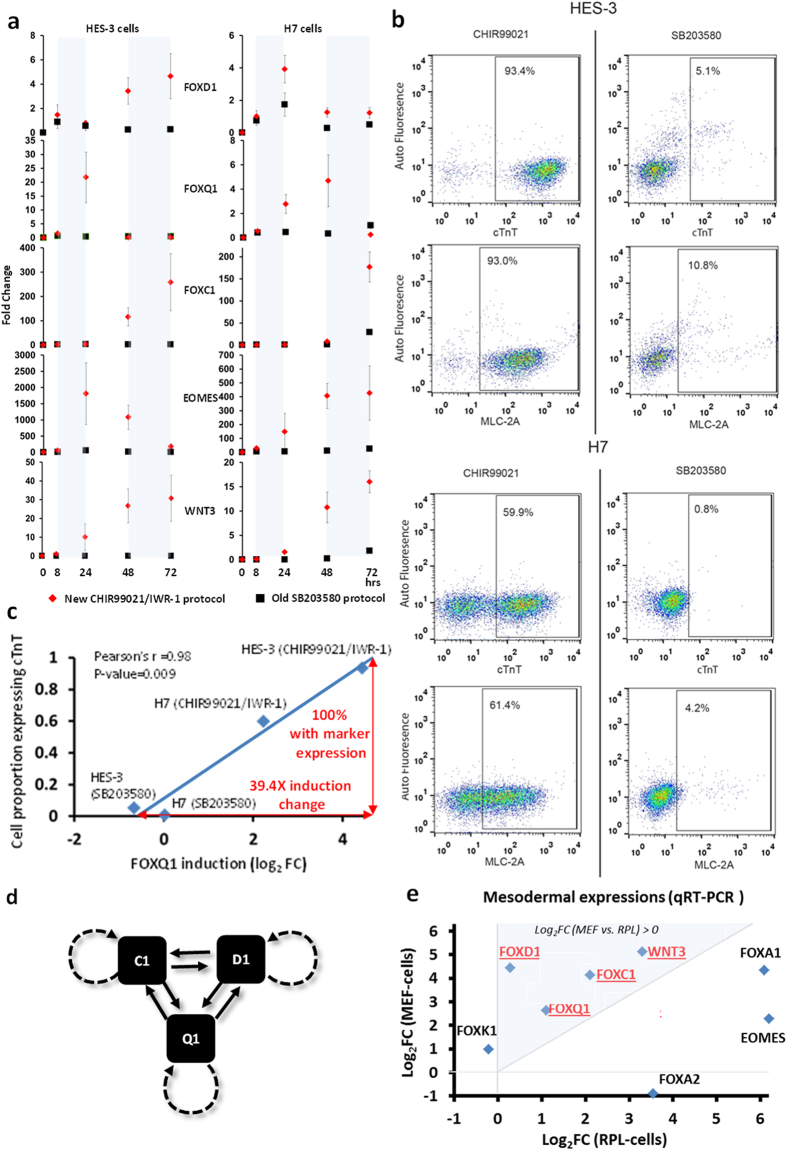
FOX inductions correlated with cardiomyogenesis efficiency of hESC cultures. (**a**) Expression profiles of FOX trio, WNT3 and EOMES in H3 cells and H7 cells after treatment with CHIR99021/IWR-1 and SB203580 protocols. (**b**) FACs analysis of anti-cTnT and anti-MLC-2A-stained HES-3 and H7 cells at day 12 after treatment with the same protocols. (**c**) Cell proportion expressing cardiac marker cTnT at various FOXQ1 induction levels upon application of differentiation protocols. (**d**) One plausible model of auto- and mutual FOX activations which was consistent with their correlation with cardiomyogenesis efficiency. (**e**) qRT-PCR validation of embryonic mesodermal expressions in HES-3 cultures. Log2 fold change level of RPL-cells and MEF-cells was in comparison to MGEL-cell baseline.

FOXC1 is known to be involved in the developing mesoderm^31^ and late-stage cardiovascular development^44^. However, what is less obvious but made clear from our work is that FOXC1, and possibly also FOXD1 and FOXQ1, have regulatory roles in promoting the mesoderm-differentiation capability of hESCs. We further deduced and demonstrated one method of FOX induction by WNT pathway activation using the CHIR99021/IWR-1 protocol. Our *in silico* ‘differential signaling analysis’ also hinted that similar signaling induced by mouse feeders enhances parental FOX expressions, thus explaining MEF-cells’ propensity for differentiation. Consistent with a close association between WNT3 and the FOX TFs, we noted their transcriptional co-regulation (Fig. 6e), similar FOX binding sites (Table 2) and co-activations by the WNT pathway (Fig. 6a), as well as shared functions as inducers of mesoderm differentiation^27^,^31^,^44^. Taken together, there may be, indeed, mechanistic and biological basis of them working together as a ‘module’ promoting cardiomyogenesis (Fig. 4). On the other hand, cardiac-specific genes were not up-regulated in MEF-cells compared to either MGEL or RPL-cells based on microarray analysis (Supplementary Information), and these included prominent NKX2-5 and MEF2C genes (Supplementary Table 2). It further reinforced that it was mesodermal factors, and not cardiac factors, which determined the cardiomyogenesis efficiency of our HES-3 cultures. From a regulatory perspective, genes either known or implicated by us to be involved in cardiomyogenesis (such as those in our regulatory model, Fig. 4), were not differentially DNA-methylated, and were thus supposedly regulated by other factors including FOX TFs.

Most importantly, the differentiation effects of FOX activations have to be verified directly, rather as a downstream consequence of CHIR99021/IWR-1–induced WNT pathway activation. To this end, an independent research group with broad interest in FOX TFs have conducted gain- and loss-of-function experiments at about the same time as our study, which clearly proved our postulated functional role of FOXC1 in promoting the cardiomyogenesis potential of embryonic stem cells^45^. After parental knockdown, embryoid bodies (EBs) displayed significant decrease in the expressions of downstream mesodermal target, T-bra, as well as final cardiac markers, Mef2C, Nkx2-5 and cTnT (Fig. 4) while over-expression resulted in EBs having markedly augmented Mef2C and Nkx2-5 expressions. The finding on cTnT is alike to our results in Fig. 6c. Functionally, while 15% of control EBs beat spontaneously 30 times per minute and all responded to external electrical stimuli, knockdown EBs had no beat rate even with stimuli. Consistently, FOXC1 over-expression in parental cells increased the proportion of beating EBs to 28% at 63 times per minute, all in synchrony with external stimuli. Thus, parental FOXC1 level was an efficacious and causative determinant of final cardiomyogenic outcome by various measures. While there is no similar data for FOXQ1 and FOXD1, we suggested their possible roles in constituting a bistable switch between the pluripotent state and the mesodermal lineage. As the conventional mesodermal markers, T-bra and MESP1^20^, were not differentially-expressed among our hESC cultures based on microarray analysis (corrected p-values > 0.7), there potentially existed a niche applicability of FOX TFs as markers of the mesoderm-differentiation capacity of hESCs. Their levels can also be used to check activation of differentiation and to correlate future course of events as we have demonstrated (Fig. 6c), and as such, gauge the effectiveness of industrial platforms. Fundamentally, FOX TFs, especially FOXC1, should be viewed as a crucial link between WNT signaling and mesodermal cell formation, driving the cells to their cardiomyogenic destiny under the correct conditions.

Going forward, more studies are required especially to validate FOXC1 expression usage with regard to differentiation into other mesodermal lineages such as erythrocytes and other muscle cells. To understand how hESCs make decision in committing to specialization, the switching behaviour of FOXC1 expression among biologically-plausible states should be further investigated by scientists familiar with stability analysis^46^, as well as loop analysis using the Routh-Hurwitz stability criterion^47^. In summary, knowledge-based genome-wide analysis of the ‘omics’ profiles of various hESC cultures allowed us to uncover the regulatory role of FOX TFs, principally FOXC1, in marking mesoderm differentiation potential. We also provided the evidence of their early expressions being strongly correlated with final cardiomyogenesis outcome upon WNT induction.

## Methods

### Cell cultures

HES-3 ([46 X,X]; ES Cell International) and H7 ([46 X,X]; WiCell Research Institute) cells were cultured using either Knockout™ SR medium (Life Technologies) or mTeSR™1 (Stemcell Technologies), following a previously described protocol^48^. For ‘omics’ profiling experiments, the ‘MEF-cells’ were HES-3 cells cultured on immortalized human fibroblast feeders, followed by mouse embryonic fibroblast feeder layer for at least 60 passages while the ‘MGEL-cells’ were the same HES-3 cells cultured on Matrigel™ (BD)-coated surfaces for at least 60 passages; ‘RPL-cells’ were HES-3 cells cultured for 57 passages on Matrigel™-coated surfaces, and subsequently re-plated and cultured on plates with mouse embryonic fibroblast feeders for another 15 passages. The medium was refreshed daily and cultures were passaged weekly. Cultures were incubated at 37 °C in a humidified atmosphere with 5% CO_2_. Cells were sent for Fluorescence-activated cell sorting analysis bi-weekly to test for pluripotent markers (*OCT-4*, *TRA-1-60* and *mAB-84*). For each growth condition, we had three cell culture replicates.

### Cardiac-directed differentiation via SB203580 small molecule

hESCs were cultured using Knockout™ SR medium, washed using phosphate buffered saline with Ca^2+^/Mg^2+^ (Invitrogen), cut into small clumps (EZ-passage tool; Invitrogen) and seeded at 1.33 × 10^6^ cells/ml as EBs into ultra-low attachment 12-well plates (Nunc). The differentiation medium was made of Dulbecco’s modified Eagle’s medium (Invitrogen) supplemented with 2 mM L-glutamine (Invitrogen), 0.182 mM sodium pyruvate (Invitrogen), 1% nonessential amino acids (Invitrogen), 0.1 mM 2-mercaptoethanol, 5.6 mg/l transferrin (Invitrogen), and 20 mg/l sodium selenite (Sigma). To induce differentiation, a p38-MAPK inhibitor solution (SB203580; Sigma), dissolved in dimethyl sulfoxide (Sigma), was added into the medium at a concentration of 5 μM. The medium was refreshed every 2 days.

### Cardiac-directed differentiation via CHIR99021/IWR-1 small molecule

hESCs were cultured using mTeSR™1 (Stemcell Technologies) in 12-well plates (Nunc) for 5 days with an initial seeding density of 1 × 10^5^ cells/cm^2^. Cells were washed using phosphate-buffered saline (PBS) with Ca^2+^/Mg^2+^ and subsequently, CHIR99021/IWR-1 (8, 15 μM) in RPMI/B27-insulin (Invitrogen) was added for the first 24 h and removed via medium change (day 0 to day 1). On Day 3 of differentiation, cells were treated to IWR-1 (5 μM; Stemgent) in RPMI/B27-insulin. IWR-1 was removed via medium change on day 5 and cells were maintained in RPMI/B27-insulin thereafter^37^. Cultures were kept at 37 °C with 5% CO_2_.

### Beating aggregate count and its correlation with cardiac expressions

At day 12, beating aggregate proportion was manually evaluated for contractility using a phase contrast microscope (EVOs, AMG). With the subsequent introduction of FACs analysis which automated identification of cardiomyocytes based on marker genes, we studied the correlation between marker expressions and beating aggregate counts in order to relate the latter legacy data to new findings in the rapidly evolving field^49^. A correlation R^2^ of 0.64 was reported for cTnT.

### Fluorescence-activated cell sorting (FACs)

Cells were dissociated into single cells with Tryple^TM^ (1x) (Invitrogen) and passed through a 40 μm cell strainer (Becton Dickinson). Subsequently, cells were fixed and permeabilized (Invitrogen) according to manufacturer’s directions and incubated with primary antibodies, anti-myosin light chain (anti-*MLC-2A*. 1:100; Synaptic Systems) and anti-troponin-T (anti-cTnT; 1:200; Thermo Scientific) for CM differentiation efficiency assessment and *OCT4* (1:100; R&D Systems), *mAB84* (1:20)^50^ and *SSEA4* (1:100; BioLegend) for hESC pluripotency assessment. Alexa Fluor 647^®^ goat anti-mouse (Invitrogen) was used as the secondary antibody. All incubations were conducted at room temperature for 30 minutes. Fluorescent measurements were done using flow cytometer (GUAVA, Millipore).

### Immunohistology

Cells were harvested on day 12 of differentiation, washed with phosphate-buffered saline (PBS) with Ca^2+^/Mg^2+^, and fixed with fixing reagent A (Invitrogen). Cryo-sectioned slides were washed twice with PBS, permeabilized and blocked by 0.1% Triton X-100 and 10% goat serum respectively. Anti-troponin-T (Thermo Scientific) antibody was used for staining cardiac specific proteins. Nuclear staining was done using Slow Fade Glow with DAPI (4′6-diamidino-2-phenylindole) (Invitrogen). The fluorescence was observed using a Nikon TI-E fluorescence microscope coupled with Nikon imaging software, NIS elements.

### Gene expression and DNA-methylation measurements

Total RNA was isolated from frozen samples (<5 × 10^6^ cells) by using Trizol (Invitrogen) and purified using the RNeasy Mini Kit (Qiagen) following the suppliers protocol. cDNA was synthesized from 1 μg total RNA using Maxima cDNA synthesis kit (Fermentas). For DNA extraction, GenElute™ Mammalian Genomic DNA Miniprep Kit was used and the manufacturer’s protocol (Sigma) was followed.

To analyze mRNA expressions, quantitative real time PCR (qRT-PCR) was performed on the 7500 Real Time PCR system using a standard two-step amplification protocol followed by a melting curve analysis. Per well, the total volume of 20 μl consisted of 1.0 μl cDNA, 10 μl TaqMan (2×) (Invitrogen) and 9.0 μl RNase-free water. qRT-PCR plates were pre-made with primers by Applied BioSystems (Supplementary Table 1). The housekeeping gene *GAPDH* was chosen as the reference gene from which we computed the relative expression levels of other genes by subtracting their average threshold cycle (Ct) from the average Ct value of *GAPDH* in the same culture. This value (ΔCt) indicates the log_2_ fold-change (FC) of each gene with respect to *GAPDH*. For expression comparison between culture conditions, the log_2_FC value for each gene is similarly given by subtracting the ΔCt value of condition from the other (ΔΔCt).

DNA samples from HES-3 cells grown on the various platforms were used for the measurement of methylation changes using the Illumina Infinium HumanMethylation27 BeadChip, RevB. At the same time, total mRNA samples were collected and sent for whole genome gene expression assay using the Illumina HumanWG-6 Beadchip. The samples were collected from the parental colony and they should be representative of the starting materials.

### Microarray data processing, normalization and quality control

The gene expression data was pre-processed using the lumi package (ver 2.2.1) available in R/Bioconductor. Given the signal intensities, we read in values that have a detection p-value threshold of 0.05. Quantile normalization was then performed on the log intensity values (base 2) across all samples. Following this, unidentified and non-gene probes are then removed, reducing the total number of probes from 38,275 to 25,435 for subsequent differential expression analysis. The DNA-methylation data was processed and the intensities were normalized using the GenomeStudio software suite (Illumina Inc). The beta-value (which is a measure of relative methylation level and ranges from 0 to 1) was calculated for each of the probes for further analysis^51^.

We inspected microarray variability and reproducibility by hierarchical-clustering (*average-link*age and *Euclidean* distance) the data. Expression data was filtered to remove probes without gene matches, and thereafter a standard deviation = 0.5 filter was applied for expression data and 0.05 for DNA-methylation data. Samples from different conditions were determined to be reliably distinguished.

To further confirm that our sample cells were truly parental hESCs, and the reliability of our microarray profiling, we looked at the expression levels of pluripotent markers^6^ (*POU5F1, NANOG, SOX2*), cardiac markers^6^ (*NKX2-5, MEF2C*), an embryonic fibroblast marker^52^ (*CD44*) and a housekeeping gene (*GAPDH*). From Supplementary Table 2, we observed consistently high expressions for the housekeeping gene and pluripotent markers, while cardiac-related and embryonic fibroblast marker genes had low levels. The observations indicated that the samples were representative of starting cells before cardiomyogenesis, rather than differentiated or contaminant mouse feeder cells. Notably, the absence of contamination can be attributed to the ability of Illumina microarray platforms to exploit differences in the 3′ UTR of human and mouse mRNAs, thus avoiding cross-species reactivity^53^,^54^.

### Differential analysis (gene expression and DNA-methylation)

To elucidate the differentially-expressed genes (DEGs), we performed a rank product test^25^ between culture conditions. Rank product is a non-parametric statistical method based on ranking the FC difference between samples. For our work, we use the online implementation RankProdIt^55^ to obtain the list of DEGs and accepted a 5% false discovery rate. The same procedure was also conducted on DNA-methylation data, with an additional beta-change requirement of 25% to select genes with the largest DNA-methylation difference among cell cultures^51^. Expression and DNA-methylation levels (beta) in RPL- and MEF-cells were given as log_2_FC and log_2_ (beta change) respectively after subtracting MGEL levels that had zero cardiomyogenesis efficiency. Subsequent comparisons were made primarily between RPL-cells and MEF-cells.

### Gene regulation definitions

‘Positive regulation’ referred to positive log_2_FC values in a comparison, and vice-versa. In this regard, ‘increased regulation’ meant that the value became more positive for positive log_2_FC, and more negative for negative log_2_FC values. Examples were log_2_FC = 2 to log_2_FC = 5, log_2_FC = −3 to log_2_FC = −7, etc. Similarly, ‘decreased regulation’ meant that the value became less positive for positive log_2_FC, and less negative for negative log_2_FC. Examples were log_2_FC = 5 to log_2_FC = 2, log_2_FC =−7 to log_2_FC =−5, etc. In the same manner, ‘reversed regulation’ meant that the value became negative for positive log_2_FC, and positive for negative log_2_FC. Examples were log_2_FC = 5 to log_2_FC = −6, log_2_FC = −9 to log_2_FC = 6. ‘Decreased/increased/reversed regulation in MEF-cells’ was in comparison to RPL-cells.

### Association test between gene expression and DNA-methylation dynamics

DEG lists from the three classes of expression dynamics were tested for association with the three classes of DNA-methylation dynamics, using the Fisher’s Exact Test for two categorical variables at two levels. Genes common to expression and DNA-methylation microarrays were used as background gene-set.

### Clustering of gene expression and DNA-methylation dynamics

K-means clustering of gene expression and DNA-methylation probe profiles were done using MultiExperiment Viewer (MeV)^56^. For each gene, DNA-methylation probes with beta changes greater than 15% were evaluated to be consistent with each other in their change direction, with the exception of probes associated with the GNAS Complex Locus.

### Knowledge-based functional, pathway and binding-site screening

High-throughput functional, pathway and ChIP-seq binding-site screening of DEGs and DMGs were performed using DAVID’s enrichment analysis (version 6.7)^57^ based on a modified Fisher’s Exact P-value (EASE score). The literature, databases and expert knowledge on pluripotent stem cells and cardiomyogenesis were involved in shortlisting and analysing functions, pathways and genes-of-interest in an iterative manner. Such knowledge-based integrative ‘omics’ approaches have been used successfully^58^,^59^. Microarray genes that can be mapped to DAVID were used as the background in enrichment analysis. In this regard, the DNA-methylation platform covered about 73% (12174/[12174+4541]) of the gene expression microarray.

### TF binding site analysis

ChIP-seq data in DAVID were obtained from the Encyclopaedia of DNA Elements (ENCODE) consortium (http://genome.ucsc.edu/ENCODE/index.html). The database featured 91 cell lines based on the binding profiles of 161 TFs. It considered a gene to be a putative target if it had TF binding site(s) within 10 Kbps 5′ upstream^45^ and 3 Kbps 3′ downstream, inclusive of introns and exons. While cell line-specific targets were modulated according to epigenetics and other cellular factors, a significant enrichment for annotated targets (>20% enrichment and P-value < 0.05) will implicate global regulation by the TF.

### Paired T-test for mesodermal and cardiac gene-sets

Tests were conducted in a pairwise manner between HES-3 cultures to evaluate their expression differences for six gene-sets. Predefined by the Gene Ontology (GO) Project (www.http://geneontology.org/) and the PANTHER classification system (http://www.pantherdb.org/), the gene-sets were (1) ‘BP00248: mesoderm development’ [PANTHER] with 484 genes, (2) ‘GO:0007498~mesoderm development’ [GO] with 69 genes, (3) ‘GO:0001707~mesoderm formation’ [GO] with 34 genes, (4) ‘GO:0048333~mesodermal cell differentiation’ [GO] with 8 genes, (5) ‘GO:0055007~cardiac muscle cell differentiation’ [GO] with 21 genes and, (6) GO:0055013~cardiac muscle cell development’ [GO] with 9 genes. For testing purpose, the average expression of a gene in one cell culture was paired with that of the same gene in a second culture. P-values for gene-set 4-6 were not significant between all pairs of hESC cultures.

### Interactome construction

DEGs were shortlisted from functional screening based on expert knowledge and then mapped onto signaling pathways from major databases^60^,^61^. The resulting pathways were overlaid with each other, augmented with knowledge from the literature, and further made concise for differential signaling analysis. In building the interactome mediating cardiomyogenesis, the binding site data of 10 FOX TFs on their enriched targets were represented as a network for the gene cluster with increased regulation. Subsequently, collective interactions among the 10 FOX TFs, potential enhancers of cardiomyogenesis in the same cluster, and signaling pathways were added from a human-specific database^62^. Biological knowledge and verified mechanisms of cardiomyogenesis were further integrated from the literature. From the interactome landscape, we looked for network motifs that were over-represented in DEGs and yet included known potent enhancers of cardiomyogenesis. The network template was then pruned to keep the most noteworthy motif in the concise context of biologically-relevant and verified interactions.

### Differential signaling analysis

We developed an in-house approach to understand the effect of differential expressions on signaling cascades affecting cardiomyogenesis. In comparing a cascade AȷB → CȷD between two conditions, the relative signal transduction effect of each component (A, B, C or D) is taken as the mathematical product of its effect on a substrate (activation ‘→’: +1; inhibition ‘ȷ’: −1) into its expression regulation (log_2_FC). A positive number for a component indicated an increased propensity for substrate activation, and vice-versa. For instance, given that log_2_FC(A) = −2, log_2_FC(B) = 0.5, log_2_FC(C) = −0.5, the effect of AȷB, B → C and CȷD is given by −1x(−2), 1x(0.5) and −1(−0.5) respectively. A series of large positive numbers that correspond to consecutive components in a cascade suggests enhanced signaling sensitivity originating from expression changes.

## Additional Information

**How to cite this article**: Yeo, H. C. *et al*. Genome-Wide Transcriptome and Binding Sites Analyses Identify Early FOX Expressions for Enhancing Cardiomyogenesis Efficiency of hESC Cultures. *Sci. Rep*. **6**, 31068; doi: 10.1038/srep31068 (2016).

## Supplementary Material

Supplementary Information

Supplementary Data

## Figures and Tables

**Table 1:** Mesodermal DEGs with increased positive regulation in MEF-cells compared to RPL-cells.

Gene	RPL-cells (log_2_ FC)	MEF-cells (log_2_ FC)	References
FOXA1	0.67 →	*1.36*	N.A.
FOXA2	*1.02* →	*2.37*	Marker of a supporting feeder^63^
*FOXC1*	0.26 →	*2.80*	Cardiovascular development^44^,^64^
*FOXD1*	*1.02* →	*1.40*	*Proposed mesoderm induction*
*FOXQ1*	0.12 →	*2.17*	*Proposed mesoderm induction*
FOXK1	0.02 →	*1.20*	N.A.
*WNT3*	0.80 →	*1.42*	Mesoderm induction^27^
*EOMES*	0.51 →	*2.24*	Cardiomyogenesis induction^33^
NODAL	*1.19* →	*1.50*	Cardiomyogenesis promoter^28^
FBLN2	0.55 →	*1.28*	Cardiac differentiation [RefSeq]
ADAM19	0.49 →	*1.29*	Role in cardiac development^65^
NKX6-2	0.37 →	*1.01*	N.A.
LBX2	0.18 →	*1.16*	N.A.
CRIP1	0.54 →	*1.18*	Regulated in cardiac development _ENREF_64^66^
AFP	0.93 →	*1.24*	Cardiac Progenitor marker [LifeMap]
TNNC1	0.33 →	*1.23*	Cardiac Progenitor marker [LifeMap]
MSX1	0.19 →	*2.09*	N.A.
MYL7	0.48 →	*1.72*	Cardiac Progenitor marker [LifeMap]

**Table 2:** FOX binding sites of (i) genes involved in mesoderm development and (ii) signaling.

Genes	FOX TF Binding Sites	Annotation
FOXA1	FOXD1, −Q1	Mesodermal gene
FOXA2	FOXD1, −Q1, −C1	Mesodermal gene
FOXC1	FOXD1, −Q1, −C1	Mesodermal gene
FOXD1	FOXD1, −Q1, −C1	Mesodermal gene
FOXQ1	FOXD1, −Q1, −C1	Mesodermal gene
EOMES	FOXD1, −Q1	Mesodermal gene
MSX1	FOXD1, Q1	Mesodermal gene
ADAM19	FOXQ1, −C1	Mesodermal gene
CRIP1	FOXC1	Mesodermal gene
EPHB1	FOXD1, −Q1, −C1	Mesodermal gene
EPHB6	FOXQ1, −C1	Mesodermal gene
FBLN2	FOXQ1, −C1	Mesodermal gene
WNT3	FOXD1, −Q1	Mesodermal gene/Mesodermal signaling (WNT)
ID3	FOXD1, −Q1, −C1	Mesodermal signaling (TGF-beta)
ZIC2	FOXD1, −C1	Mesodermal signaling (Hedgehog)

**Table 3:** Major signaling cascades identified with significant difference in expression and hence signaling propensity^*,+^.

Cascade	MEF vs. RPL (Log_2_FC)						Average
BMP	*BMP2*→	*BMPR2*→	*SMAD4*→	*ID1*→	*Mesoderm Induction*		
	+(1.75)	+(1.05)	+(0.49)	+(1.18)			1.12
NODAL	*NODAL*→	*SMAD4*→	*EP300*→	*FOXC1* →	*Mesoderm Induction*		
	+(0.31)	+(0.49)	+(0.61)	+(2.54)			0.99
WNT	*WNT3*→	*FZD3*→	*FRAT2*→	*AXIN2*→	*LEF1*→	*FOXC1*^+^	
	+(0.62)	+(0.94)	+(0.57)	+(0.41)	+(1.08)	+(2.54)	1.03
FGF	*FGF2*→	*FGFR3*→	*RRAS*→	*FOXC1*→	*Mesoderm Induction*		
	+(0.81)	+(2.03)	+(−0.46)	+(2.54)			1.23
Hedgehog	*ZIC2*→	*GLI2*→	*FOXC1*→	*Mesoderm Induction*			
	+(0.99)	+(0.65)	+(2.54)				1.39

^*^Log_2_ Fold Change is multiplied by +1 for substrate activation ‘→’, and −1 for inhibition ‘−’ in evaluating signaling propensity. ^+^Mesoderm Induction follows.

**Table 4:** Pearson correlation coefficient between maximum FOX TFs induction (72 hours) and proportion of cells expressing cardiac markers at day 12 of cardiomyogenesis.

	FOXC1		FOXD1		FOXQ1	
	Correlationcoefficient	One-tailedP-value	Correlationcoefficient	One-tailedP-value	Correlationcoefficient	One-tailedP-value
cTnT	0.86	0.070	0.90	0.051	0.98	0.009
MLC-2A	0.85	0.076	0.89	0.056	0.98	0.011

Sample size n = 4 (SB203580, HES-3; SB203580, H7; CHIR99021/IWR-1, HES-3; CHIR99021/IWR-1, H7).
