# Supplementary material for: Genome-Wide Transcriptome and Binding Sites Analyses Identify Early FOX Expressions for Enhancing Cardiomyogenesis Efficiency of hESC Cultures
Source: Sci Rep. 2016 Aug 9;6:31068. doi: 10.1038/srep31068 (PMC4977469; doi:10.1038/srep31068)
Supplement: Supplementary Information [file srep31068-s1.doc]

# Supplementary Information

**Genome-Wide Transcriptome and Binding Sites Analyses Identify Early FOX Expressions for Enhancing Cardiomyogenesis Efficiency of hESC Cultures**

Hock Chuan Yeo, Sherwin Ting, Romulo Martin Brena, Geoffrey Koh, Allen Chen, Siew Qi Toh, Yu Ming Lim, Steve Kah Weng Oh, Dong-Yup Lee

**Supplementary Figure 1** DNA-methylation dynamics after K-means clustering using dot-product metric.


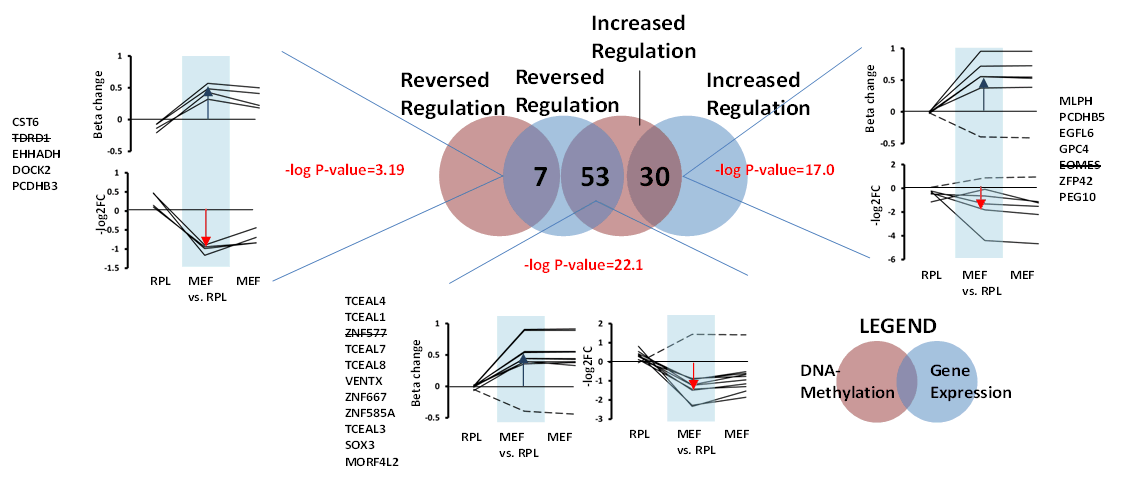


## Supplementary Figure 2. Significant statistical associations between gene expression and DNA-methylation dynamics based on Fisher’s Exact test-of-independence.

As illustrated, 20/23 sampled genes had the expected opposite direction of regulation for gene expression and DNA-methylation in MEF vs. RPL comparison. The three genes with strikethrough symbol did not have the expected relationship between expression and DNA-methylation. Trends as depicted by filled lines were those of positive DNA-methylation changes and negative expression changes while broken lines indicated negative DNA-methylation changes and positive expression changes


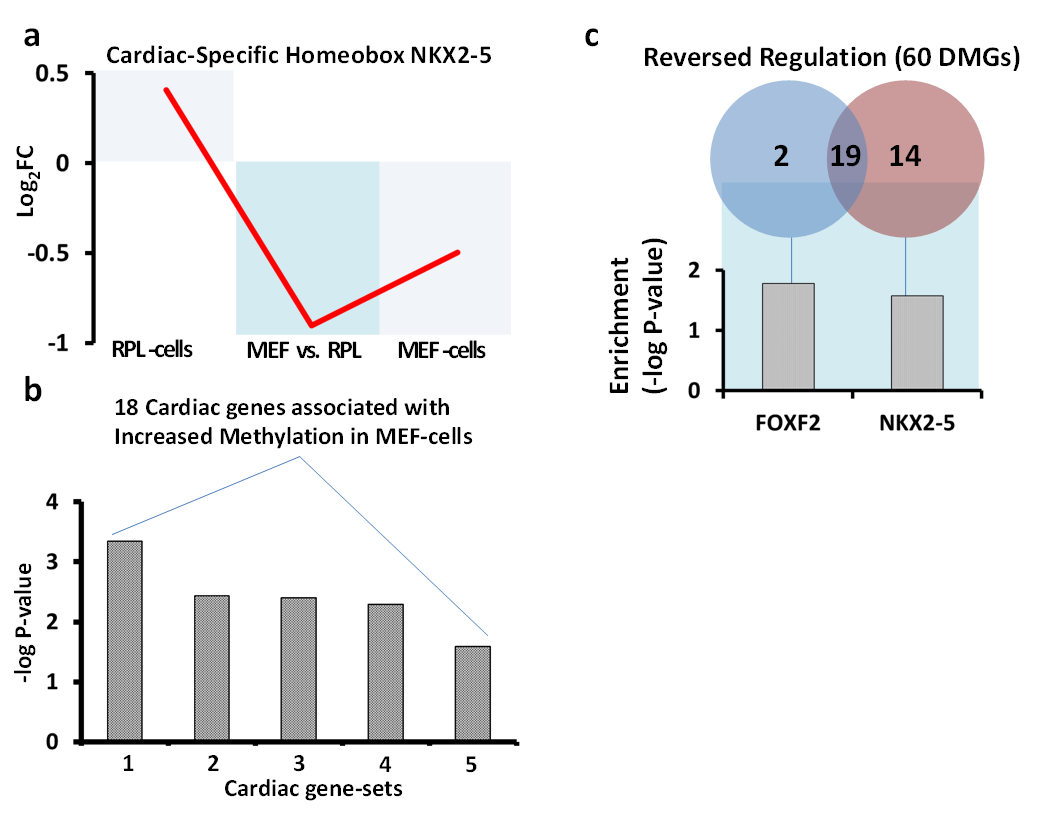


## Supplementary Figure 3. Cardiac-specific gene expression levels in cardiomyogenesis-efficient MEF-cells

(a) Higher microarray expression of Cardiac-Specific Homeobox NKX2-5 in RPL-cells compared to MEF-cells. (b) Cardiac gene-sets were associated with increased DNA-methylation in MEF-cells. Cardiac gene-set 1 referred to ‘hsa04260:Cardiac muscle contraction’ (KEGG_PATHWAY); cardiac gene-set 2 referred to ‘786696:ATP synthase, H+ transporting, mitochondrial F1 complex, alpha subunit 1, cardiac muscle’ (BIND); cardiac gene-set 3 referred to ‘779357:actin, alpha, cardiac muscle 1’ (BIND); cardiac gene-set 4 referred to ‘cardiac muscle’ (SP_PIR_KEYWORDS); cardiac gene-set 5 referred to ‘GO:0008016~regulation of heart contraction’ (GOTERM_BP_FAT). (c) 33 DMGs with reversed regulation in MEF-cells were annotated NKX2-5 targets.

## Supplementary Table 1. Assay IDs of qRT-PCR primers provided by Applied BioSystems.

| **OFFICIAL Gene Symbol** | **Assay ID** |
| --- | --- |
| EOMES | Hs01015629_m1 |
| FOXA1 | Hs00270129_m1 |
| FOXA2 | Hs00936490_m1 |
| FOXC1 | Hs00559473_s1 |
| FOXD1 | Hs00270117_s1 |
| FOXK1 | Hs01595620_m1 |
| FOXQ1 | Hs00536425_s1 |
| GAPDH | Hs99999905_m1 |
| WNT3 | Hs00229135_m1 |

## Supplementary Table 2. Average log_2_ expression levels of housekeeping and marker genes under different culture conditions.

| **Gene** | **MGEL (Average)** | **RPL (Average)** | **MEF (Average)** |
| --- | --- | --- | --- |
| **Housekeeping Genes** | | | |
| GAPDH | 15.55 | 15.59 | 15.46 |
|  | | | |
| **Pluripotent Stem Cell Markers** | | | |
| POU5F1 (OCT4) | 14.58 | 14.79 | 14.75 |
| NANOG | 12.26 | 13.02 | 12.87 |
| SOX2 | 14.64 | 13.74 | 14.06 |
| **Mouse and human**  **embryonic fibroblast marker** | | | |
| CD44 | 8.68 | 9.22 | 8.97 |
|  |  |  |  |
| **Cardiac Cell Markers** | | | |
| NKX2-5 | 8.88 | 9.29 | 8.38 |
| MEF2C | 8.13 | 8.18 | 8.19 |

# Supplementary Text

## Effect of DNA-methylation change on gene expression of hESCs

- Overall, DNA-methylation profiles were durable. The 133 genes whose DNA-methylation have responded newly to re-plating, constituted less than 1% of all genes represented on the microarray (top and bottom panel, Supplementary Figure 1). The other 425 DMGs (middle panel, Supplementary Figure 1) were understandably due to prolonged differences between mouse feeder and Matrigel environments. Even so, these are only 3% of all genes.
- Using a similar gene enrichment approach[^10^](#_ENREF_10) that elucidated the regulatory relationship between TF activities and target gene expressions, we determined significant association between some of the gene expression and DNA-methylation classes:
- Genes with increased DNA-methylation regulation in MEF-cells were highly enriched in genes with (i) increased expression regulation in MEF-cells (-log P-value=17.0) and, (ii) reversed expression regulation in MEF-cells (-log P-value=22.1) (Supplementary Figure 2).
- Genes with DNA-methylation regulation reversal were also associated with genes with expression regulation reversal (-log P-value=3.19).
- The statistical associations were meaningful biologically. About 87% (20/23) of tested genes had, as expected, opposite direction of DNA-methylation changes compared to gene expression changes in MEF vs. RPL comparison (Supplementary Figure 2).Screening based on the DAVID database did not reveal significant functions related to cardiomyogenesis for associated DMGs (Supplementary Data).
- Genes that had responded in their DNA-methylation after re-plating (bottom panel, Supplementary Figure 2) were not statistically associated with differentially-expressed genes (P-value =0). However, their expressions during initiation of cardiomyogenesis may still be modulated by their different DNA-methylation status. 8 of these DMGs were also associated with mesoderm development (P-value < 0.01) and 49 were annotated as FOXC1 and/or –D1 targets (P-value=3.8x10^-6^, 1.34x10^-5^ respectively). Hence, their changes may affect mesoderm differentiation and hence final cardiomyogenesis outcome.

**No evidence of cardiac programs enhancement in MEF-cells**

Besides no significant up-regulation of two cardiac gene-sets based on paired T-test (Methods), there were three other findings that suggested cardiac programs were not enhanced in MEF-cells:

(i) higher NKX2-5 expression in RPL-cells over MEF-cells from microarray profiling (Supplementary Figure 3a),

(ii) increasing DNA-methylation from MGEL- to RPL- to MEF-cells (middle panel, Supplementary Figure 1) for 5 cardiac gene-sets for a total of 18 genes (Supplementary Figure 3b) and,

(iii) heightened methylation of a gene cluster with 33 NKX2-5 targets (top panel, Supplementary Figure 1) in MEF-cells over RPL-cells (Supplementary Figure 3c).

### References

1 Sang, N. *et al.* MAPK signaling up-regulates the activity of hypoxia-inducible factors by its effects on p300. *J Biol Chem* **278**, 14013-14019, doi:10.1074/jbc.M209702200 (2003).

2 Woo, C. W., Lucarelli, E. & Thiele, C. J. NGF activation of TrkA decreases N-myc expression via MAPK path leading to a decrease in neuroblastoma cell number. *Oncogene* **23**, 1522-1530, doi:10.1038/sj.onc.1207267 (2004).

3 Zhu, J., Blenis, J. & Yuan, J. Activation of PI3K/Akt and MAPK pathways regulates Myc-mediated transcription by phosphorylating and promoting the degradation of Mad1. *Proc Natl Acad Sci U S A* **105**, 6584-6589, doi:10.1073/pnas.0802785105 (2008).

4 Li, J. *et al.* CBP/p300 are bimodal regulators of Wnt signaling. *Embo J* **26**, 2284-2294, doi:10.1038/sj.emboj.7601667 (2007).

5 Zhang, S. *et al.* Wnt/beta-catenin signaling pathway upregulates c-Myc expression to promote cell proliferation of P19 teratocarcinoma cells. *Anat Rec (Hoboken)* **295**, 2104-2113, doi:10.1002/ar.22592 (2012).

6 Kuwahara, A. *et al.* Wnt signaling and its downstream target N-myc regulate basal progenitors in the developing neocortex. *Development* **137**, 1035-1044, doi:10.1242/dev.046417 (2010).

7 Nishihara, A. *et al.* Role of p300, a transcriptional coactivator, in signalling of TGF-beta. *Genes Cells* **3**, 613-623 (1998).

8 Frederick, J. P., Liberati, N. T., Waddell, D. S., Shi, Y. & Wang, X. F. Transforming growth factor beta-mediated transcriptional repression of c-myc is dependent on direct binding of Smad3 to a novel repressive Smad binding element. *Mol Cell Biol* **24**, 2546-2559 (2004).

9 Serra, R., Pelton, R. W. & Moses, H. L. TGF beta 1 inhibits branching morphogenesis and N-myc expression in lung bud organ cultures. *Development* **120**, 2153-2161 (1994).

10 Yeo, H. C. *et al.* Integrated transcriptome and binding sites analysis implicates E2F in the regulation of self-renewal in human pluripotent stem cells. *PLoS One* **6**, e27231, doi:10.1371/journal.pone.0027231 (2011).
